# A Proposal for Addressing Bioethical Concerns Along the 10‐Step Framework for Community Engagement

**DOI:** 10.1111/hex.70345

**Published:** 2025-07-09

**Authors:** Abdou Simon Senghor, Michelle Medeiros, Claudia Baquet, Franklin Lance, C. Daniel Mullins

**Affiliations:** ^1^ University of Maryland School of Pharmacy Baltimore Maryland USA; ^2^ Department of Practice, Sciences, and Health Outcomes Research University of Maryland, Baltimore Baltimore Maryland USA; ^3^ Mount Lebanon Baptist Church Baltimore Maryland USA

**Keywords:** bioethics, collaboration, community‐engaged research, community–academic partnership, ethical deliberation, health equity

## Abstract

**Introduction:**

Building trust by applying an ethical deliberation approach can increase Black/African American participation in clinical and translational community‐engaged research (CEnR).

**Materials and Methods:**

We provide examples of case studies from the literature to identify ethical issues associated with each step of the 10‐Step Framework. To address these ethical issues, we applied an ethical deliberation approach embodied in three moments: (1) broadening and deepening the understanding of the situation and/or research scenarios, (2) envisioning action scenarios for more trustworthy research, and (3) coming to a judgement based on the comparative evaluation of scenarios.

**Results:**

Examples of ethical issues identified include a lack of shared decision‐making on proposed research topics, lengthy periods for data analysis and reporting that frustrate partners who want more timely results, and the lack of privacy, confidentiality and non‐compliance with consent permissions for the dissemination of results.

**Conclusion:**

We recommend tailoring the ethical deliberation approach to any project dealing with ethical issues and conducting empirical studies to test this approach in CEnR.

**Patient or Public Contribution:**

As part of a community‐engaged research (CEnR) project, this paper benefited from the contribution of F.L., a member of a community organisation, as a co‐author. This author was actively involved in contributing to the review of ethical issues in community‐engaged research and how the ethical deliberation approach can contribute to addressing these ethical issues.

## 1 Introduction‐Background

Health disparities and inequities in healthcare are barriers to improving the health of disadvantaged populations, particularly Black/African American populations, in the United States [1, 2]. Disparities are noted in the specific context of enrollment in clinical trials where Black/African American populations are under‐represented [3, 4]. In this paper, we propose an ethical deliberation approach that researchers, policymakers, community leaders and Black community members can use to solve ethical problems using the PATIENTS Program 10‐Step Framework for continuous engagement (10‐Step Framework). Because the 10‐Step Framework aims to promote engagement in research, interest in Black/African American under‐representation in clinical trials stems from the historical context of harm suffered during the Tuskegee Syphilis Study, which remains a contemporary source of mistrust of physicians [5]. In addition, researchers report that less than 20% of clinical trials provide information about the treatments and side effects for Black people [6, 7]. To reduce these disparities, community‐engaged research (CEnR) can facilitate a more inclusive and responsive approach to address patient and community needs and concerns [8, 9, 10], foster trust [9, 10, 11], and increase trustworthiness of clinical trialists and in research protocols and practices [9, 10].

However, there are challenges related to the feasibility of community interventions due to structural racism; in addition, some interventions have difficulty achieving effect within communities because consideration was not given to the factors that influence community health behaviours [12]. Indeed, public health interventions often involve policy changes that create barriers to assigning communities to comparison conditions. In addition, the feasibility of using randomised controlled trials (RCTs) is challenged by unintended side effects.

To address ethical issues related to CEnR, solutions in the form of rules, guidelines or frameworks [13, 14] have been proposed, but these solutions do not take into account the decisions of stakeholders at each stage of the research process.

## 2 A Need for Patient and Community Engagement Perspective to Address Ethical Challenges in CEnR

Defined as ‘the process of working collaboratively with and through groups of people affiliated by geographic proximity, special interest, or similar situations to address issues affecting the well‐being of those people’ [15, 16], CEnR is conducted to address the mistrust between researchers and communities and to encourage the recruitment of diverse community perspectives [10]. The concept of community is defined in terms of its systemic, social, virtual and individual understandings. From a system's standpoint, a community consists of ‘different parts that represent specialized functions, activities, or interests, each operating within specific boundaries to meet community need’ [15]. The social definition of a community emphasises its constitution as social and political networks that link individuals, community organisations and leaders.

A community can be understood from a virtual perspective, where individuals and community organisations with a common interest organise and interact via the Internet to inform each other and make decisions that affect their lives. A community can also be understood from an individual perspective, in which a community is defined by whether or not the individual belongs to that community. The individual may belong to several communities, or may change communities, which may affect their participation in the activities of that community [15]. From this perspective, a community may not participate in research activities if it does not feel that its interests or needs are being addressed.

CEnR is often focused on project‐specific outcomes, making it difficult to have a broader impact [17]. Community partners and academics may not have the same priorities or the same goals [18, 19]. The community may not understand the approach used by the research, which is a barrier to their participation [19]. For some researchers, patient engagement is a threat that encourages them to hand over their research to patients; furthermore, it is difficult to get patients to participate in research because the funder has often already chosen the topic [20]. Additionally, ethical training is only focused on the researcher's perspective or, when related to community engagement, ethical concerns are addressed through the lens of ethical principles [21].

## 3 Materials and Methods

### 3.1 The Literature Review

For the purposes of this study, we provided examples of case studies from the literature that were chosen because they involved ethical challenges related to one or more steps of the 10‐Step Framework.

### 3.2 The 10‐Step Framework

Initially conceptual [8], the 10‐Step Framework emerged from prior theory and application of community‐based participatory research and community action approaches [22, 23, 24] as well as the emergence of patient‐centred comparative effectiveness research under the Affordable Care Act of 2010. Studies pointed out the need to bridge the gap between community‐based research and an expanded role for patients as co‐developers of research rather than merely participants [25, 26]. A PCORI‐funded report on rare diseases mentioned this gap and noted that approaches to engagement were not well established [25]. These studies on patient and stakeholder engagement did not provide solutions for engaging patients and stakeholders in earlier research stages, such as prioritising research topics or formulating patient‐centred outcomes research (PCOR) questions, compared to later stages, such as translating and disseminating evidence [25, 26, 27], preceded the development of the 10‐Step Framework with the aim of proposing ways to co‐develop patient‐centred research. However, these approaches were more community‐based than focused on patients' experiences, preferences and values.

The 10‐Step framework is similar to an earlier UK National Institute for Health Research Patient and Public Involvement initiative focusing on comparative effectiveness and PCOR [28, 29]. The initiative made recommendations on how to engage patients and the public throughout the research process. The UK model consists of eight steps that promote patient involvement in research. The UK model consists of eight steps: (1) identifying and prioritising issues, (2) designing studies, (3) developing grant proposals, (4) undertaking and managing projects, (5) analysing and interpreting results, (6) disseminating findings, (7) implementing appropriate methods, and (8) monitoring and evaluating projects. Furthermore, patient public involvement should not cause a power imbalance between patients, clinicians and stakeholders. No topics are off‐limits, and patients can be involved in choosing scientific literature to be integrated into the research process [30]. The 10‐Step Framework establishes a horizontal relationship that enables full patient involvement.

The PATIENTS Program 10‐Step Framework aims to promote the involvement of diverse patients and stakeholders in clinical research, as defined by Mullins, Abdulhalim and Lavallee [8]. The 10 steps of the framework are: (1) Topic solicitation, (2) Prioritisation, (3) Framing the question, (4) Selection of comparators and outcomes, (5) Creation of conceptual framework, (6) Analysis plan, (7) Data collection, (8) Reviewing and interpreting results, (9) Translation and (10) Dissemination. These steps are defined according to their purpose in the context of patient engagement. Subsequently, empirical studies were carried out to improve the performance of this framework. For example, Edwards et al. [31] proposed the Patient Engagement Translation Table (PETT), which maps patient engagement methods to the 10‐Step Framework. Sofolahan‐Oladeinde et al. [20] conducted a qualitative study to assess the usefulness of the framework in terms of patient engagement. In another study, Sofolahan‐Oladeinde et al. [32] highlight the relationship between the 10‐Step Framework, community engagement principles, and PCOR principles (trust, honesty, transparency, co‐learning, reciprocal relationships, partnerships and respect) to show how the nine Community‐Based Participatory Research (CBPR) principles can be applied to patient engagement in the different phases (pre‐engagement, ongoing engagement and sustained engagement) of a PCOR study. Among the recommendations, the authors suggest creating a collaborative work environment to avoid tensions related to the differences in goals and priorities between researchers and community partners. The 10‐Step Framework was also used to promote patient engagement in pragmatic oncology clinical trials [33].

The 10‐Step Framework is more than just a conceptual model. It has been used in operational studies that demonstrate the impact of continuous engagement. In a study examining patient preferences in thyroid cancer treatment using clinical vignettes, Kim et al. [34] used the 10‐Step Framework to establish a community advisory board composed of patients, advocates, caregivers and community‐based endocrinologists to develop and improve clinical vignettes. These clinical vignettes, which clearly describe treatments for low‐risk thyroid cancer, may make patients more likely to choose less invasive treatment options over traditional surgical resection. In a different context, Pogorzelski et al. [35] used the 10‐Step Framework to ensure continuous patient engagement in the PREP‐IT trials to explore participants' experiences with trial participation. The PREP‐IT trials consist of two multicentre cluster‐randomised crossover trials Aqueous‐PREP (Pragmatic Randomized Trial Evaluating Pre‐Operative Aqueous and Antiseptic Skin Solution in Open Fractures) and PREPARE (Pragmatic Randomized Trial Evaluating Pre‐Operative Alcohol Skin Solutions in Fractured Extremities). The authors report high levels of participant satisfaction, as well as strong enrollment and retention rates, which the 10‐Step Framework helped achieve. The 10‐Step Framework was also used to develop the Value in Hepatitis C Virus Treatment Patient‐Centered Model [36]. A stakeholder advisory board (SAB), including four patients with Hepatitis C Virus, three infectious disease specialists, one general practitioner, two pharmacists and a national patient advocacy organisation representative, take part in the study. This study using a patient‐centred SAB based on the 10‐Step Framework emphasises cost‐saving in treatment decisions when patient and stakeholder engagement is effective.

To optimise the use of the 10‐Step Framework, it is important to consider the ethical aspects of patient engagement. This will help researchers and clinicians better integrate the needs, experiences, values and requirements of patients participating in clinical research. Thus, the objective of this study is to apply an ethical deliberation approach to the 10‐Step Framework to address ethical concerns at each step.

The application of an ethical deliberation approach to the 10‐Step Framework is a contribution to the work done by Black scholars specialising in bioethics, such as those involved in the report ‘A Critical Moment in Bioethics: Reckoning with Anti‐Blackness through Intergenerational Dialogue’ published by the Hastings Center Report [37]. Thus, this manuscript contributes to the transformation of bioethics from its role in perpetuating structural injustice to its obligation to promote social justice and advance health equity [37]. The application of this ethical deliberation approach to the 10‐Step Framework is also based on the fact that both the approach and the framework are based on patient and stakeholder engagement.

### 3.3 The Ethical Deliberation Approach

The ethical deliberation approach [38], to which we referred, is offered by pragmatist ethical theory, especially in the writing of John Dewey, an American philosopher. The ethical deliberation approach we propose is based on Dewey's pragmatism, with an emphasis on the concepts of transaction and experience. According to Dewey

An experience is always what it is because of a transaction taking place between an individual and what, at the time, constitutes his environment, whether the latter consists of persons with whom he is talking about some topic or event, the subject talked about being also a part of the situation; or the toys with which he is playing; the book he is reading (in which his environing conditions at the time may be England or ancient Greece or an imaginary region); the materials of an experiment he is performing [39].

Through this transaction between the agent and its environment, transformative knowledge emerges, enabling the agent to grow [40]. Such a process could serve as a basis for understanding interactions during the deliberative process. Thus, the process could evolve into the choice of an ethical solution based on shared experience, enabling the agents to grow and deliberate according to shared values. In the deliberative process, the agent shares the experience gained from previous situations, but also from the experiences of other agents. According to Dewey, the transaction produces knowledge through the experience born of the agent's relationship with his environment. Thus, from a pragmatist's point of view, deliberation is the result of shared experiences that ultimately enable deliberating agents to grow and propose solutions based on shared values.

Methodologically, the ethical deliberation approach we propose follows a process of enquiry, examining a problematic situation, engaging in deliberation and ultimately evaluating the ethical merits of different courses of action [38]. Based on the previous evaluation by Senghor and Racine [38] of the quality of an ethical deliberation, the approach is embodied in three moments: (1) broadening and deepening the understanding of the situation, (2) envisioning action scenarios, and (3) coming to a judgement based on the comparative evaluation of scenarios. Moment 1 begins with the recognition of situations as morally problematic [38]. Morally problematic situations refer to ‘situations in which our typical habits of behaviour, that is, typical ways of doing things and solving everyday problems, are challenged in ways that relate to morality’ [41]. Moment 2 mentions possible scenarios for solving ethical problems, and Moment 3 refers to the selection of scenarios according to their ethical merits.

Following the logic of improving the 10‐Step Framework for better promotion of patient engagement, we propose that this ethical deliberation approach will result in the identification of ethical issues at each step and the proposal of scenarios respecting stakeholders' values. The only deliberative approaches we found in the literature are limited to specific ethical issues that focus on issues other than the conduct and process of clinical research. For example, Gillies, Elwyn and Cook [42] focus their study on the measurement of participation in clinical trials through a comparison of the deliberations of trial participants with those of non‐participants. Ethical issues were also addressed, and solutions proposed using moral case deliberation in a clinical context [43]. Our study uniquely applies the ethical deliberation approach, which we propose involves stakeholders identifying ethical problems at each step of the 10‐Step Framework and proposing an ethical solution corresponding to each ethical problem.

## 4 Results

### 4.1 Ethical Challenges Associated With the 10‐Step Framework for Continuous Engagement

Our experiences applying the 10‐Step Framework revealed there are ethical challenges with each step. We have identified some of these ethical challenges and present them in Table 1.

**Table 1:** Case study examples at each step from the literature.

Steps of the 10‐Step Framework	Bioethical issues or challenges	Mechanism to address the challenges	Examples
Topic solicitation	Power imbalance, slanted towards academic researchers, does not allow for balanced shared decision‐making on topic generation.	Facilitate effective community participation in topic selection, with community leadership organisations as equal partners, to ensure that the topic selected is important to community members in that it addresses their health needs. Ensure that participants are diverse.	The non‐governmental organisation (NGO) Alternatives for Community and Environment (ACE) in the low‐income Roxbury section of Boston reached out to Harvard University's School of Public Health and other potential partners to study and address the high rates of asthma in their neighbourhood. Although having a community partner, such as ACE, identify an issue and catalyse a research partnership may be the ideal, it is often the privileged researcher who initiates a CBPR project [32].
Prioritisation	No shared decisions or consideration of community members' values/views about the priorities.	Establish fair processes that ensure transparency, accountability and understanding of a particular community. Promote community empowerment, that is, a commitment to reduce power differentials between community members and researchers.	Example 1: In responding to a call for proposals, this requires that either a partnership already exists or that time and resources be available to bring potential partners together to decide on these key issues. Unfortunately, this is often not the case, and researchers may have to approach potential community partners after decisions have already been made regarding the research priorities [33]. Example 2: ‘Biases can arise in biomedical research priority‐setting due to disease‐based lobbying from different stakeholder groups and government bodies. This can result in priorities being selected for funding that are not always priorities that the community deems most important. Rare diseases can be sidelined in biomedical research priority‐setting in favour of diseases that are more common within a geographic community in order to address the needs of a greater number of people.’ [34]
Framing the question	Institutional procedures limiting the participation of community members in the formulation of the question	Developing a memorandum of agreement (MOA) to help researchers and the community discuss and agree on roles, responsibilities and expectations in CEnR to prevent overburdening communities.	In CBPR, the community often comes up with the research question or issue of interest based on personal experience, but in a randomised controlled trial (RCT), the funding agency or investigator generally develops the question based on pressing health issues identified from surveillance or other data sources [35]. The Prevention Research Centers at the University of New Mexico conducted an RCT on obesity prevention with 16 rural Head Start centres across the state. An RCT conducted in the traditional way is done in an artificial ‘laboratory’ setting within an academic health centre or practice setting; an RCT in the community setting can be just as rigorous but with more flexibility and community participation [35]. The challenge has been to develop strategies to engage the community in the research process within a short period of time and with clear communication and agreement [35]
Selection of comparators and outcomes	Lack of consideration of community members' and/or patients' prioritised concerns or decisional dilemma when assessing comparators and related outcomes	Follow an equal and rigorous procedure in the treatment of control and experimental groups and make measurements within the same time frame. Develop transparent, lay outline of risks and benefits of comparators and related outcomes. Incorporate decisional dilemma factors when applicable.	Studies conducted in a small community health centre led to a close relationship between participants. Participants assigned to different treatment arms are likely to know each other, mention their experiences to others in the waiting room, or talk about their music therapy experiences while attending other treatments at the clinic [36]. When control participants hear about participants' experiences in the music therapy sessions, they may feel resentful for not having been assigned to that group [36].
Creation of a conceptual framework	The difference in the definition of concepts used by community members and researchers is a barrier to development or participation.	In the process of creating the conceptual framework, promote the community–academic partnership throughout, including updates and the drafting and proposing of recommendations.	In the late 1990s, the West Virginia University PRC and its partners were conducting research on teen smoking cessation in North Carolina, largely among white teens. The researchers saw tobacco as the problem, but many community members did not. This was a significant issue to resolve before the project could move forward. A major breakthrough occurred when the partners reached a declarative insight that *tobacco addiction*, not tobacco, was the challenge to be addressed [35].
Analysis plan	Lengthy process of the analysis of data creates frustrations among partners who view it as slowing down the process [37]. Time constraints due to other obligations of the partners [37].	Provide transparent communication about the analysis plan and its timing from the beginning, and give periodic updates during the analysis. Consider leveraging other professionals (e.g., consultants) with additional bandwidth, which may enable them to carry out project activities more quickly.	‘The active involvement of all partners in the research process, including questionnaire development, survey administration, and feedback and interpretation of data, exacts a tremendous commitment of time from all participants.’ [37]
Data collection	Questions related to privacy, confidentiality, who has access to identifiable data and what is done with the data after the project	Develop clear and concise data collection plans which delineate the following: specific data elements (when possible include rationale), timing of collection, access controls, incorporation of community contributions, plans (if any) for data use after project and commitment to disseminating results.	Wearable devices that measure exposures, behaviours (e.g., physical activity and sleep patterns), health data (e.g., heart rates) and location, often linked to the internet, are increasingly commonplace and raise ethical concerns about who has access to the data and for what purposes, as well as privacy and informed consent [38].
Reviewing and interpreting results	Difference in prioritisation of outcomes among community members, community organisations and health professionals.	Suggestions included promoting the use of methods appropriate to the research goal and the context and interests of the community, using qualitative data to assess the context and process of community research interventions, and triangulation involving multiple data sources, methods and investigators to establish data reliability.	Within a given community, men and women and youth may all identify different concerns, and community members, community leaders and health professionals may all identify different priority health concerns. Interpretation of these differences and decisions about how to integrate and prioritise the results is a challenge for community partners and researchers alike [37].
Translation	Issues related to community members' preferences and researchers' goals to achieve high‐level changes. Research can violate the privacy of the community members who want to keep information private.	Promoting the use of formal or informal research protocols and memoranda of understanding to address the ethical issues associated with translation in CEnR, including values related to respect for community members' preferences, privacy and confidentiality, and ensuring that community members' needs are met.	In the Grandparent Caregiver Study done in Oakland, California, during the crack cocaine epidemic in the early 1990s, women expressed concern that one of the smaller findings could do harm to the community should it become public, particularly if it came to the attention of the conservative state governor [39].
Dissemination	Lack of understanding of research findings shared by researchers due to poor science and research communication	Provide a give‐back of the findings to the community in lay terms via the avenue(s) preferred by the community (e.g., infographic, social media and printed flyers). Encouraging phase‐by‐phase dissemination rather than waiting until the end of the project.	Timing of dissemination can also flame tensions if researchers want to wait to release results after peer‐reviewed publication, but community members want results made public sooner to advocate for action [38]. Example of a photovoice project that focuses on the sharing of photos with policymakers and others as a means of affecting broader level change. The homeless participants in the Language of Light project were eager to share their photos with policymakers, but they were not interested in using this or other venues to press for policy‐level change [39].

### 4.2 Using an Ethical Deliberation Approach to Solve Bioethical Issues Related to the 10‐Step Framework

In this section, we apply an ethical deliberation approach to the 10‐Step Framework to explain how this should be done before the research project starts. We suggest that at each step, we identify examples of ethical issues and apply ethical deliberation. Participants in this process include researchers, clinicians, community members, leaders of Black/African American community organisations, and Black/African American patients. The diversity of stakeholders' backgrounds is important in an ethical deliberation process, as mentioned by Senghor and Racine [38], who developed the ethical deliberation approach used in this study. Collaborative diversity enables stakeholders to learn from each other, to assess morally problematic situations together and to address them through shared experiences.

#### 4.2.1 Moment 1: Broadening and Deepening the Understanding of the Situation

Participants in the ethical deliberation session must first work to identify bioethical issues for and with the community. This collaboration among participants is one way to engage in a process that promotes ethical community engagement by addressing the needs of the community and building trusted partnerships [44]. Community members may not have the same experiences when invited to participate in clinical research. Similarly, community‐engaged researchers may have unique experiences with community members when recruiting for clinical research. Thus, it is critical to encourage diversity in deliberation sessions [38] to elicit as diverse and rich a range of bioethical concerns as possible. Participants need to focus their propositions on what constitutes a bioethical problem for the community in clinical research participation. This orientation offers a different perspective from bioethics, which has often sought to address ethical issues and assess risks at the individual level [45]. Each participant must identify bioethical issues and share them with the other participants. The reference to the community is important because focusing on bioethical concerns at the individual level can cause harm to the community if inappropriate solutions are proposed to address these ethical issues [45]. For example, when identifying ethical issues related to topic solicitation, clinicians should not influence or impose their topic on community members because the topics are important to them. Stakeholders need to value each other's experiences, opinions and preferences so that all ethical issues are considered. Once all the ethical issues for each step have been clearly identified and collected, the stakeholders must select one ethical issue that is a priority for the community and whose resolution could promote health equity. During the deliberation sessions, participants must be open, transparent and honest [44] to clarify the prioritised ethical issues. Failure to do so can lead to misidentification and misplacement of priorities, resulting in less effective ethical solutions [46]. This prioritisation is important because it enables participants to engage in a fruitful deliberative process.

#### 4.2.2 Moment 2: Envisioning Action Scenarios

Once the priority for each step has been identified, the participants propose possible scenarios for resolving the problematic situation. For each step, the proposed scenarios are adapted to the values of the participants. For example, an ethical problem related to the dissemination of results that harm the community must take into account the scenarios proposed by the community members based on their values. In this process of gathering scenarios, participants' powerful scenarios must be consistent with the character of responsive communitarianism, integrating both the values of the community and those of the participant [47]. The openness of the participants will ensure that a participant does not have the right to dictate scenarios that, even if based on scientific evidence, may cause harm to the community. Nor should researchers' scenarios that are considered to have a scientific basis be imposed on community members' scenarios, which may be based on experience. Respect for each participant's knowledge can be promoted in this way to reduce deliberative inequality [48] based on the fact that, for example, researchers have more knowledge about clinical research. Scenarios must be treated equally, and participants must behave in a way that does not create a power relationship that favours participants' scenario suggestions over those of their peers. The example of ‘yarning’ as an approach that facilitates communication, idea development and equitable partnerships in the context of community engagement can be one option for eliciting scenario proposals from participants. Yarning is a cultural form of communication used by Indigenous people in Australia. It fosters respectful relationships and allows participants to appropriate the research topic in a way that meets the needs and expectations of the community [49]. In addition, the involvement of Black academic researchers in deliberative sessions can promote the proposal of scenarios that better reflect the values of the community. In this way, tendentious decisions on the basis of a social construction of race can be avoided [50].

#### 4.2.3 Moment 3: Coming to a Judgement Based on the Comparative Evaluation of Scenarios

In this third moment, town hall participants discuss which of the proposed scenarios for each step is the most feasible. Participants also discuss which of the scenarios respects the values of the community and which is the least harmful of the scenarios for each step. The focus on the scenario's minimal harm to the community constitutes the ethical merit on which the most ethical scenario is based. The prospect of a scenario that is deemed satisfactory by all stakeholders, without imposition or influence due to hierarchical rank, favours greater consideration of the voices of community members and the valorisation of their experience and culture. Integrating these aspects could help to support ethical decision‐making, as is reflected in a study by Parker et al. [51] in which three expert panels were selected to participate in a research Ethics Training for Health in Indigenous Communities. The panellists identified culturally based tools and tools related to Indigenous approaches to support ethical decision‐making that respect the moral values and culture of indigenous populations and communities. To avoid conflicts among stakeholders, it is important to integrate cultural elements into the decision‐making process. In the Netherlands, a moral case deliberation study by Inguaggiato et al. [52] based on the case of a 32‐year‐old male rugby player of Moroccan–Dutch descent with a poor neurological diagnosis led the medical team to withdraw his treatment. Initially determined to keep their loved one alive, the family and the medical team agreed to stop the treatment thanks to the discussion between the family and the intercultural healthcare consultant. This example illustrates the importance of considering the cultural factors and their influence in the ethical deliberation process. Conducting ethical deliberation also requires epistemic humility [53] on the part of participants to avoid imposing a scenario that is harmful to the community, even if the proposal suggested to the participants is based on scientific data. For example, a clinician who imposes a scenario from a study whose target population did not include under‐represented groups to resolve an ethical issue related to the prioritisation of issues runs the risk of dictating issues to members of communities who do not share the same priorities.

### 4.3 Methodological Applications of the Ethical Deliberation Approach

Having shown how each step of the 10‐Step Framework could be considered in the three moments of deliberation, it is important to outline the methodological applications of the ethical deliberation approach. To do this, we have drawn on examples of studies dealing with ethical situations. Martinez‐Martin et al. [54] used a modified Delphi technique to identify the ethical issues associated with digital phenotyping for mental health applications. Stakeholders reached a consensus on the recommendations they made to address these ethical issues. By using the Delphi technique to test the ethical deliberation approach, a community‐engaged research context can help to address ethical issues related to each step of the 10‐Step Framework, as Black stakeholders in CEnR discuss and find solutions by considering the values of the community. Focus groups are another method that would help address the ethical issues associated with the 10‐Step Framework. If focus groups are designed to help stakeholders deliberate, they can achieve their goal of considering Black communities' values. A study by Rothwell et al. [55], which conducted deliberative discussion focus groups, suggested that before the focus groups, participants should be educated and informed about the topic of discussion and a topic expert co‐moderator should be invited. This approach allows participants to refine their opinions. In CEnR, knowledge of the topic of discussion is critical [56]. For instance, before engaging Black stakeholders through the three moments of the ethical deliberation process, informing and educating them and having facilitators throughout the process could help them deliberate at each step of the 10‐Step Framework by aligning their solutions with the values of the Black community. World Café methods can also be used to achieve ethical deliberation in the context of CEnR. It was used in a study by Street et al. [57] to help 84 older Australians address ethical challenges associated with the use of smart technology. The world café method consisted of discussions that were analysed by a qualitative researcher. Through the three moments of the ethical deliberation approach, we can analyse the data to understand how Black stakeholders come to a deliberation by analysing the discussions between stakeholders. The photovoice method can also be used in an ethical deliberation process, providing solutions to health problems through images and photos shared by stakeholders, as shown by Downey et al. [58]. Through the application of the methods cited as examples, CEnR, by its dialogical and collaborative nature [59], can contribute to the application of the ethical deliberation approach. Community engagement with experts on the issues under discussion can promote informed decisions [59]. This makes community engagement an important element of ethical deliberation for the participation of Black communities in clinical research. Indeed, racial concordance increases the willingness of Black people to participate in research when they work with Black clinicians [60].

## 5 Conclusion

This article offers a way to mitigate ethical challenges by applying an ethical deliberation approach based on three moments to the 10‐Step Framework for community engagement. Because this approach to ethical deliberation in a CEnR context is a theoretical proposition, further studies are needed to test it, more fully; however, we provide case study examples where researchers appear to have addressed ethical challenges associated with community engagement along the 10‐Step Framework. We also provide examples of methodological applications that could help researchers apply the ethical deliberation we propose to the 10‐Step Framework in a practical way.

## Author Contributions

**Abdou Simon Senghor:** conceptualisation, writing – original draft, methodology, writing – review and editing, formal analysis, project administration. **Michelle Medeiros:** writing – review and editing, supervision. **Claudia Baquet:** writing – review and editing, supervision. **Frank Lance:** writing – review and editing. **C. Daniel Mullins:** writing – review and editing, supervision.

## Ethics Statement

The authors have nothing to report.

## Consent

The authors have nothing to report.

## Conflicts of Interest

The authors declare no conflicts of interest.

## Data Availability

Data sharing is not applicable to this article as no datasets were generated or analysed during the current study.
